# Unveiling the Hidden Culprit: A Case of a Temporal Lobe Abscess Presenting With Nonspecific Symptoms

**DOI:** 10.7759/cureus.83954

**Published:** 2025-05-12

**Authors:** Javed A Hussain, Lokeshwar Singh, Gregory Pugh

**Affiliations:** 1 Emergency Medicine, Peterborough City Hospital, Peterborough, GBR

**Keywords:** burr hole aspiration, mri brain, otitis media, ring enhancing, temporal lobe abscess

## Abstract

A brain abscess is a potentially life-threatening condition in which a collection of pus forms in the brain tissue due to an infection. Mastoiditis and chronic otitis media are among the leading causes of brain abscesses; however, with modern advancements in imaging technology and antibiotic treatments, the incidence of such cases has significantly declined. This report describes a 72-year-old patient with no co-morbidities and a recent travel history presenting with respiratory symptoms, a short duration of ear symptoms, and confusion, who was subsequently diagnosed to have a temporal lobe abscess.

## Introduction

A brain abscess is a focal intracranial infection that may present with mild, vague symptoms or as a life-threatening emergency [1]. In the past, chronic otitis media and mastoiditis were the most common underlying etiologies; however, complications of these infections have decreased in incidence with improvements in diagnostic modalities and antibiotic therapy [2].

Brain abscesses may arise from parameningeal infections, such as otitis media, sinusitis, and mastoiditis [3]. Otogenic brain abscesses are one of the most significant life‐threatening complications of otologic infections. Given their low prevalence, otogenic brain abscesses require a high index of suspicion for diagnosis [4]. The treatment of brain abscess is challenging. Small brain abscesses have been treated empirically with antibiotics. Patients presenting with rapidly progressive neurological deficits due to the mass effect of the neuroradiologically verified brain abscess are candidates for urgent decompression [4]. Prognosis is variable and dependent on the primary source of infection or predisposing factors to brain abscess, the age of the patient, underlying disease or immune status, and previous use of antibiotics [5].

Here, we describe a patient who presented to our emergency department with symptoms of an upper respiratory tract infection and an ear infection with a new history of acute confusion. After undergoing evaluation, the patient was diagnosed with a brain abscess, which was treated with antibiotics and surgery.

## Case presentation

A 72-year-old female with no underlying health conditions presented to the emergency department complaining of a productive cough, runny nose, and shortness of breath for the past 10 days, with symptoms worsening over the last 3. Two days before her presentation to our emergency department, she had flown from the USA to the UK, during which she experienced bilateral ear popping. After arriving in the UK, she developed bilateral greenish watery ear discharge, a feeling of fullness in her ears, and hearing loss in both ears. She consulted her General Practitioner, who suspected otitis externa and prescribed her an ear spray containing dexamethasone, neomycin sulfate, and acetic acid. Her husband also noticed a change in her behavior and confusion over the 24 hours leading up to her visit, as well as brief episodes where she appeared to be stuporous. Aside from asthma, she was a healthy individual with no other comorbid conditions. She stated that she has not travelled to any countries besides the United States. She also reports no history of ear surgeries, ear infections, or open water swimming.

On examination, the vitals were a heart rate of 106 beats per minute, blood pressure of 141/83 mm of Hg, temperature of 37.8 °C, and oxygen saturation of 96& on room air. The chest was clear, and a neurological examination did not reveal any focal neurological deficits. Kernig’s and Brudzinski’s tests were performed and yielded negative results. Ears showed no signs of redness, warmth, or mastoid tenderness, although green serous discharge was appreciated from the left ear. The bilateral external auditory canal was patent, filled with discharge, and the left tympanic membrane was perforated. The tuning fork tests revealed no detectable hearing loss.

Blood investigations revealed an elevated C-reactive protein (Table 1), and the chest X-ray showed no abnormality. The patient was started on IV Co-Amoxiclav 1.2 gm and IV fluids and was admitted under the medical team with an otorhinolaryngologist consultation with a working diagnosis of suspected sepsis with the source of infection being the ear. Blood cultures were obtained and showed no growth after five days of incubation.

The ENT examination showed a posterior inferior perforation of the left ear but no findings suggestive of mastoiditis. Given the history of confusion and the suspected source of infection being otitis media, a plain CT head was performed to exclude any intracranial pathologies. Given her presentation, meningitis and encephalitis were included in our differential diagnosis, and a CT head scan was required to rule out any contraindications to performing a lumbar puncture.

**Table 1:** Serum laboratory results on the first and last days of admission

Parameter	Reference range and units	First day of admission	Last day of admission
WBC count	4-11 × 10^9^/L	11.1	7.9
Haemoglobin	115-165 g/L	134	80
Platelet count	150-400 × 10^9^/L	440	130
Lymphocytes	1.4 – 4.8 x 10^9^/L	1	1
Neutrophils	1.8 – 7.7 x 10^9^/L	9	6.5
Monocytes	0.1 – 0.8 x 10^9^/L	1.1	0.4
Eosinophils	0.1 – 0.6 x 10^9^/L	0	0
Basophils	0 – 0.1 x 10^9^/L	0	0
Sodium	133-146 mmol/L	143	138
Potassium	3.5-5.3 mmol/L	4.1	3.6
Chloride	95-108 mmol/L	95	99
Creatinine	45-84 umol/L	39	46
CRP	<5 mg/L	192	4
Lactic acid	0.5-2 mmol/L	0.99	0.6

**Figure 1:**
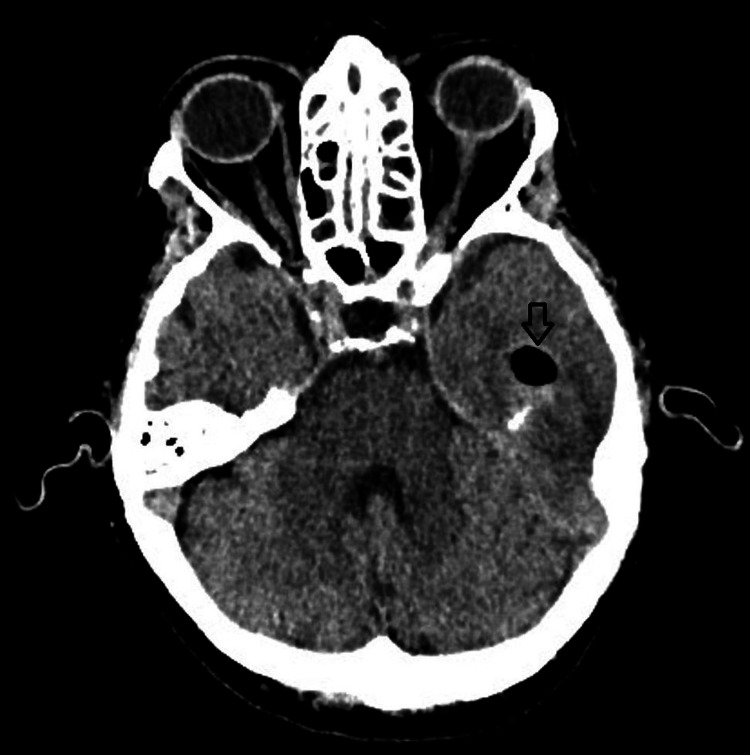
A plain CT scan of the brain showing a 12mm air-filled cyst in the left temporal lobe with mild surrounding white matter edema

**Figure 2:**
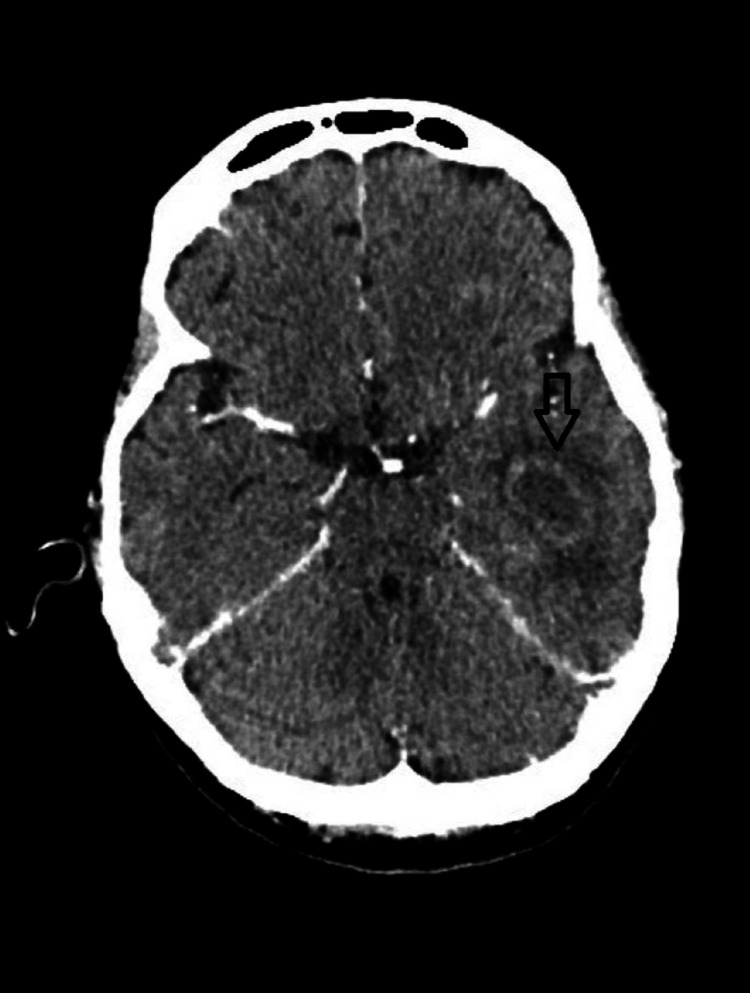
A CT scan of the brain with contrast medium showing a 3 x 1.7 cm ring-enhancing abscess in the left temporal lobe

A CT head demonstrated extensive opacification of the bilateral middle ears with a possible erosion of the left tegmen tympani along with a 12 mm air-filled cyst in the left temporal lobe with mild surrounding white matter edema, suggesting a possibly resolved abscess (Figure 1). Subsequently, a CT head with contrast was obtained, which showed a 3 cm x 1.7 cm ring-enhancing abscess in the left temporal lobe along with bilateral mastoiditis with otitis media (Figure 2).

**Figure 3:**
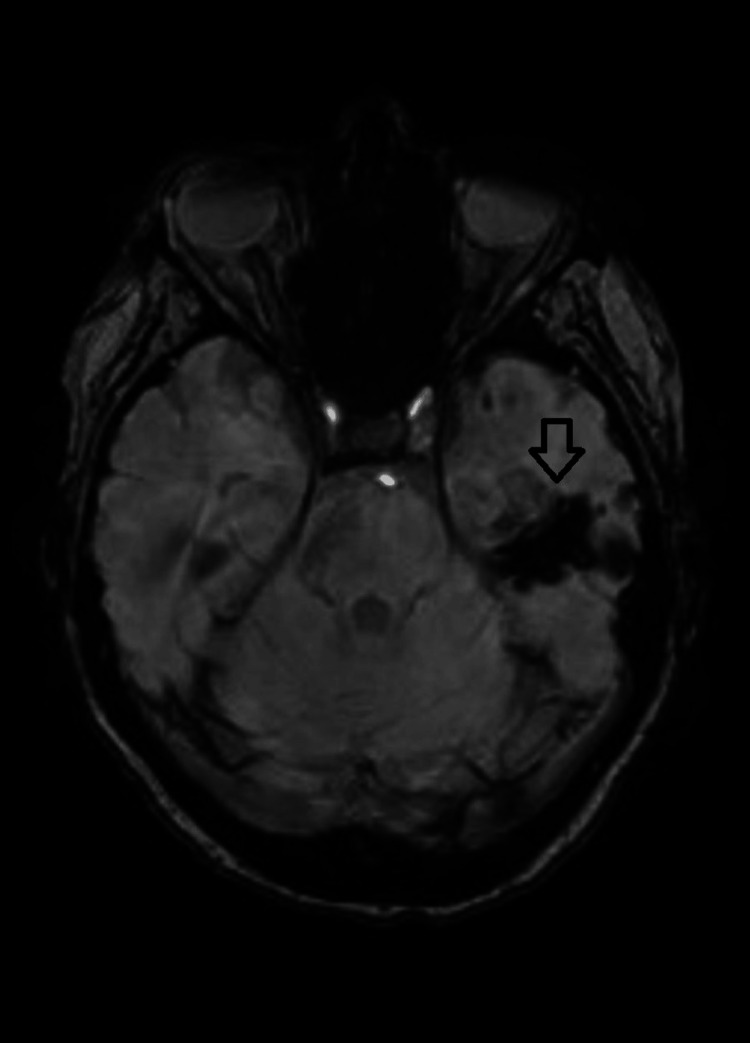
Magnetic resonance image with contrast done four weeks post-surgery showing the healing progress of the abscess and no recollection

The patient was referred to the neurosurgery team and underwent a burr hole aspiration followed by left myringotomy and grommet insertion. The patient's MRI post-surgery was satisfactory with no definite loculated collection/abscess, and she was started on four weeks of IV ceftriaxone. The cultures of pus obtained from the abscess showed Streptococcus pneumoniae. A repeat CT head with contrast was done two weeks after surgery, which showed post-surgical changes with no intra- or extra-axial enhancing collection. An MRI brain with contrast was done after four weeks of IV antibiotics to look for residual collection; it showed altered signal intensity in the left temporal lobe, suggestive of postoperative/postinflammatory changes (Figure 3). The patient improved clinically and was discharged home after her course of antibiotics.

## Discussion

The most common symptoms of a brain abscess include headache, focal neurological deficits, altered mental status, fever, seizures, nausea, vomiting, and nuchal rigidity. However, our patient presented with altered mental status but did not exhibit any of the other typical signs. Although ear infections are common, the changes in her behavior and episodes of stupor led us to consider a brain abscess, prompting further investigation. Although ear infections are common, it's crucial to maintain a low threshold for further investigation in any patient presenting with an ear infection along with the symptoms described above.

There are four stages in brain abscess formation: early cerebritis (day 1-3), late cerebritis (day 4-9), early encapsulation (day 10-13), and late capsule stage (day 14 onwards) [6]. During the early stages of brain abscess formation, the non-contrast CT head may not be able to pick out the hypodense areas. As in our case, the non-enhanced CT did not give us a definitive diagnosis; however, a contrast-enhanced CT clearly demonstrated the temporal lobe abscess. Magnetic resonance imaging (MRI) with contrast is generally considered the imaging modality of choice for diagnosing brain abscesses due to its superior sensitivity and specificity. Nevertheless, when MRI availability is limited, contrast-enhanced CT scans can serve as a reliable alternative, facilitating prompt diagnosis and treatment.

A meta-analysis by Bodilsen et al. (2023) gives evidence supporting surgical intervention. It included a total of 17 studies and found an overall case-fatality rate of 172 of 704 (24%) in conservatively treated patients compared with 140 of 1484 (9%) in those who had their brain abscess aspirated or excised. There was a significantly high recurrence rate found among the non-surgically treated patients with lesions <2.5 cm [7]. 

In a study conducted by Darlow et al. (2020) on a UK cohort of brain abscess patients, a low prevalence of Staphylococcal infections was observed, with Streptococcal species being the predominant pathogens. The predominant pathogen currently identified in this setting is penicillin-sensitive Staphylococcus intermedius [8]. Our patient's pus obtained from the abscess grew Streptococcus pneumoniae, which is a relatively rare finding based on the available data.

In a study conducted by Bodilsen et al. (2023), the most common predisposing conditions for brain abscess were dental infections in 91 out of 485 cases (19%), ear, nose, and throat infections in 67 cases (14%), previous neurosurgery in 54 cases (11%), and distant infections in 31 cases (6%) [9]. Our patient developed a brain abscess secondary to otitis media.

The combination of metronidazole and a third-generation cephalosporin is recommended for treating abscesses arising from otitis, mastoiditis, or sinusitis, while vancomycin combined with a third-generation cephalosporin is advised for other types of abscesses [10]. This antibiotic regimen was also employed in the management of our patient.

## Conclusions

This case report indicates that many patients with an underlying brain abscess may not exhibit clear symptoms, but the diagnosis should always be considered when a patient presents with ear-related symptoms. Emergency medicine clinicians must remain vigilant, as they are often the first healthcare providers to assess these patients. The lack of symptoms can make identifying the root cause challenging, but prompt and aggressive treatment is crucial for managing a brain abscess effectively. Additionally, selecting the appropriate imaging modality is crucial for detecting the abscess, with contrast-enhanced imaging playing a vital role in accurate diagnosis. Evidence indicates that surgical aspiration yields superior outcomes compared to conservative management with antibiotics, despite the size of the abscess.

## References

[1] Klein RS (2024). Brain abscess. The Merck Manual of Diagnosis and Therapy.

[2] Carpenter J, Stapleton S, Holliman R (2007). Retrospective analysis of 49 cases of brain abscess and review of the literature. Eur J Clin Microbiol Infect Dis.

[3] Yakut N, Kadayifci EK, Karaaslan A (2015). Braın abscess due to Streptococcus intermedius secondary to mastoiditis in a child. Springerplus.

[4] Duarte MJ, Kozin ED, Barshak MB (2018). Otogenic brain abscesses: a systematic review. Laryngoscope Investig Otolaryngol.

[5] Muzumdar D, Jhawar S, Goel A (2011). Brain abscess: an overview. Int J Surg.

[6] Sharma BS, Gupta SK, Khosla VK (2000). Current concepts in the management of pyogenic brain abscess. Neurology India.

[7] Bodilsen J, D'Alessandris QG, Humphreys H (2024). European Society of Clinical Microbiology and Infectious Diseases guidelines on diagnosis and treatment of brain abscess in children and adults. Clin Microbiol Infect.

[8] Darlow CA, McGlashan N, Kerr R, Oakley S, Pretorius P, Jones N, Matthews PC (2020). Microbial aetiology of brain abscess in a UK cohort: prominent role of Streptococcus intermedius. J Infect.

[9] Bodilsen J, Duerlund LS, Mariager T (2023). Clinical features and prognostic factors in adults with brain abscess. Brain.

[10] Hakan T (2008). Management of bacterial brain abscesses. Neurosurg Focus.

